# Microglia in brain aging: An overview of recent basic science and clinical research developments

**DOI:** 10.7555/JBR.37.20220220

**Published:** 2024-02-26

**Authors:** Haixia Fan, Minheng Zhang, Jie Wen, Shengyuan Wang, Minghao Yuan, Houchao Sun, Liu Shu, Xu Yang, Yinshuang Pu, Zhiyou Cai

**Affiliations:** 1 Chongqing Medical University, Chongqing 400042, China; 2 Department of Neurology, Chongqing General Hospital, Chongqing 400013, China; 3 Chongqing Key Laboratory of Neurodegenerative Diseases, Chongqing 400013, China; 4 Department of Neurology, the First Hospital of Shanxi Medical University, Taiyuan, Shanxi 030001, China; 5 Department of Gerontology, the First People's Hospital of Jinzhong, Jinzhong, Shanxi 030009, China

**Keywords:** microglia, brain aging, Alzheimer's disease, Parkinson's disease, Huntington's disease

## Abstract

Aging is characterized by progressive degeneration of tissues and organs, and it is positively associated with an increased mortality rate. The brain, as one of the most significantly affected organs, experiences age-related changes, including abnormal neuronal activity, dysfunctional calcium homeostasis, dysregulated mitochondrial function, and increased levels of reactive oxygen species. These changes collectively contribute to cognitive deterioration. Aging is also a key risk factor for neurodegenerative diseases, such as Alzheimer's disease and Parkinson's disease. For many years, neurodegenerative disease investigations have primarily focused on neurons, with less attention given to microglial cells. However, recently, microglial homeostasis has emerged as an important mediator in neurological disease pathogenesis. Here, we provide an overview of brain aging from the perspective of the microglia. In doing so, we present the current knowledge on the correlation between brain aging and the microglia, summarize recent progress of investigations about the microglia in normal aging, Alzheimer's disease, Parkinson's disease, Huntington's disease, and amyotrophic lateral sclerosis, and then discuss the correlation between the senescent microglia and the brain, which will culminate with a presentation of the molecular complexity involved in the microglia in brain aging with suggestions for healthy aging.

## Introduction

Aging is a biological process characterized by a range of structural and functional changes that occur over time^[1]^. Because of the improved lifestyles and better access to healthcare, people are living longer, and so the population is aging. Globally, there were 727 million people aged 65 and older as of 2020^[2]^. This number is expected to rise to more than 1.5 billion by 2050^[3]^. This demographic shift will also be accompanied by a significant increase in the number of age-related diseases, such as Alzheimer's disease (AD), cardiovascular disease, diabetes, and osteoarthritis^[4]^. Aging affects almost all organs in the body, including the brain. Memory, attention, processing speed, and cognitive functions tend to decrease with brain aging. Additionally, the brain becomes susceptible to injury, and its cognitive function declines. From the perspective of micro changes in an aging brain, the brain suffers from disruptions of mitochondrial homeostasis, accumulation of oxidized macromolecules, impaired signaling in response to stress, disturbances in energy metabolism, impaired DNA repair, aberrant neural network activity, inflammation, *etc.*^[5]^.

Neuroinflammation is a response to immune stimuli in the brain or the spinal cord, which is generally caused by cytokines, chemokines, reactive oxygen species (ROS), and secondary messengers. Most of these mediators are produced by the resident glia (microglia and astrocytes)^[6]^. Microglia are the focal point for any discussion of neuroinflammation, responsible for primary immune surveillance of the central nervous system (CNS). Indeed, much of the innate immune capacity of the CNS is mediated by microglia^[7]^. Microglia have been widely studied in many diseases^[8]^, and they often have a dual role in being both neurotoxic and neuroprotective in the process of aging^[9]^, which is correlated with different functional phenotypes of the microglia*.* However, the mechanisms responsible for these distinctions are not yet known. In this review, we summarize current knowledge about the roles of microglia in brain aging and provide an understanding of the mechanisms that link microglia and aging.

## The nature, role, and function of microglia

The brain consists of several different cell types, most of which originate from neural stem cells within a developing CNS. These cells include neurons, glia (oligodendrocytes and astrocytes), and microglia. Microglia, which account for 5% to 10% of all resident macrophages^[10]^, arise from C-KIT^+^/CD41^+^ erythromyeloid progenitor cells in the yolk sac^[11–13]^. In humans, the microglia renew slowly at a median rate of 28 percent per year, and some can live for over 20 years, unlike most immune cells that live for only a few days or perhaps weeks^[14]^. Throughout the brain, the microglia act as primary immune cells and are among the first to respond to pathological conditions. Innate immune receptors in microglia can directly respond to the damage-associated molecular patterns or pathogen-associated molecular patterns, and thus protect against injurious stimuli.

In a healthy and homeostatic CNS, microglia surveil the microenvironment using their sensors, promote neurogenesis and perform maintenance on synapses. Microglia coordinate with neurons, astrocytes, oligodendrocytes, and border-associated macrophages in concert, which are crucial for maintaining homeostasis. For example, the microglia help neurons migrate and survive by promoting neurogenesis and eliminating extra neurons^[15]^. In vascular remodeling, the microglia influence blood vessel development and endothelial cell elimination. Furthermore, microglia are active regulators of the blood-brain barrier's permeability and repair as a part of the glia limitans^[16]^. In the brain, astrocytes and microglia work closely together, which is needed for astrocytic formation and immune responses^[17–18]^. Aside from this, the microglia are necessary for oligodendrocyte precursor cell survival, maturation, myelination, and myelin turnover throughout the lifecycle^[19]^. Synaptic remodeling, which is essential for memory and neuronal circuits, is accomplished by microglial pruning, synaptic element displacement, and dendritic spine creation *via* neurotrophic factor release, cytokines, or neuromodulators^[20–21]^.

**Figure 1:**
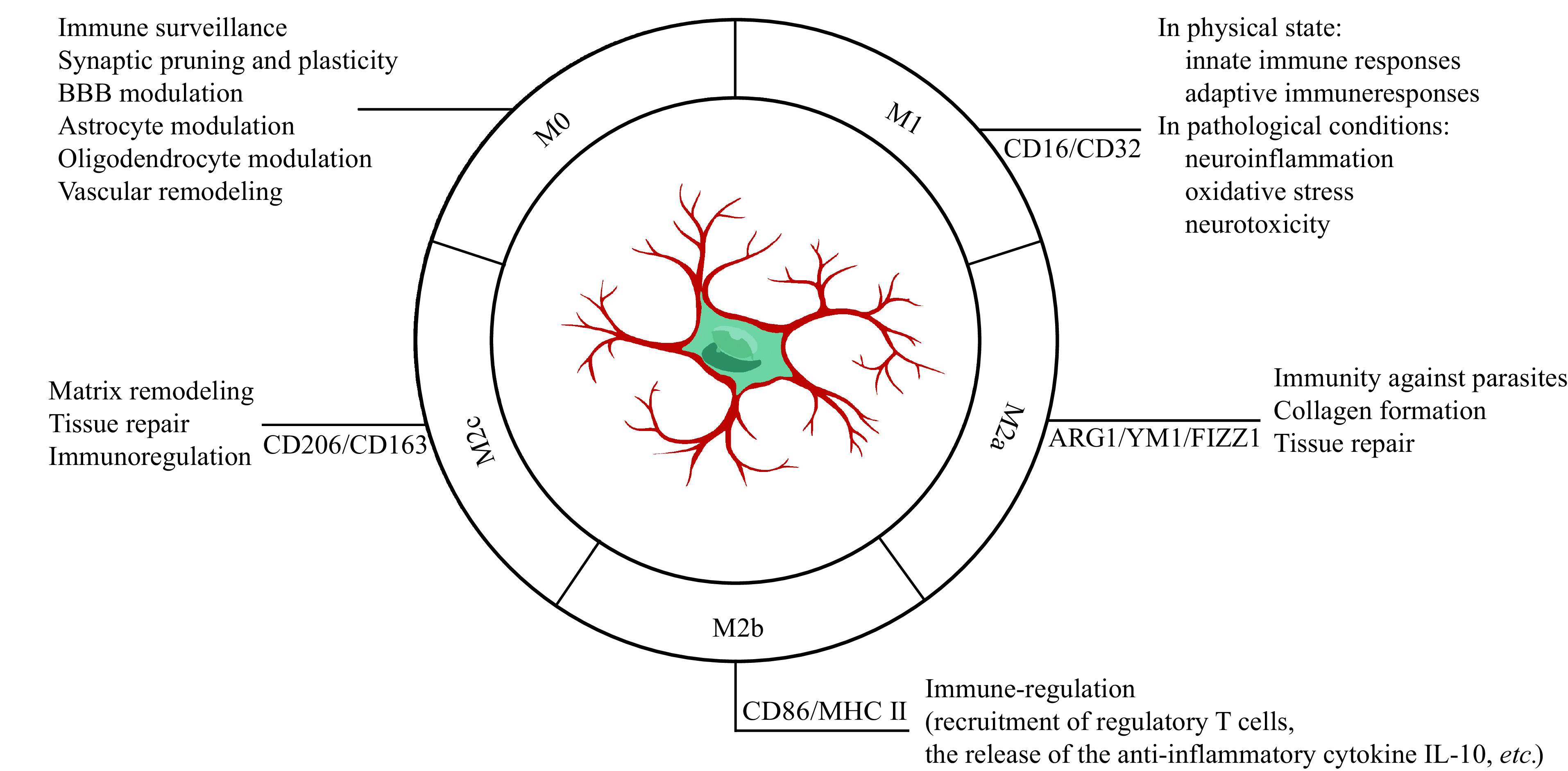
Microglial phenotypes and functions.

The plasticity of microglia has fascinated neuroscientists for many years, and the attempts to classify functional phenotypes continue^[7,22]^. In a resting state, the microglia are typically branched, spider-like structures with multiple processes and a small soma, which is known as M0 (***Fig. 1***). As soon as the microglia are activated, their morphology rapidly changes, within minutes. Typically, the microglia present an amoeboid phenotype, in which the arms are thicker, and the somata appear larger. Based on their activation status, microglia are divided into M1 (traditional activation) and M2 (alternative activation) phenotypes^[23]^ (***Fig. 1***). In the M1 phase, these microglia release a large number of pro-inflammatory cytokines by stimulating lipopolysaccharide or interferon-gamma. These then play a vital role in activating innate immune responses to combat invading infections and in activating adaptive immune responses.

However, persistent activation in pathological situations contributes to neuroinflammation, oxidative stress, and neurotoxicity. In the M2 phase, microglia release neuroprotective cytokines, such as interleukin-4/interleukin-13 (IL-4/IL-13) to promote inflammation resolution and tissue repair^[24]^. Nonetheless, it has become apparent that a single cohesive M2 phenotype fails to reflect the diversity of microglial populations, leading to the development of M2 sub-classifications. In this classification, the classic alternative activation phenotype is M2a, dealing with innate immunity, collagen formation, and repair/regeneration, and M2b, playing roles in immune regulation, as well as M2c, providing neuroprotection (***Fig. 1***). These cells have overlapping biochemical roles but differ in the activating stimuli, marker expressions, and mechanisms of action. However, it has been argued that even these subcategories cannot adequately represent the diverse array of microglial responsive states, because these classifications fail to take into account potential overlaps. Furthermore, it has been demonstrated that their phenotypes are exceedingly complicated *in vivo,* and this distinct phenotypic delineation only occurs *in vitro*, and the terminology of microglial polarization impedes rather than helps research progress^[25]^. In the end, it is essential to achieve the harmonization of microglial subtypes using new technology like multi-omic single-cell analysis. Longitudinal human and animal studies are also needed to verify the dynamic complexity of microglial phenotypes in the processes of different diseases.

## Microglia in healthy brain aging

The term "healthy brain aging" refers to brain aging without neurological diseases, which is not a pathological condition, but rather a natural process^[26]^. As with other organ systems, neuroinflammation occurs as the brain aging and is associated with the activation of microglia as well as changes at both cellular and molecular levels^[27]^. There is no doubt that aging itself is one of the leading causes of neurodegenerative diseases^[28]^. Microglia are known to play a key role in influencing neurodegenerative diseases, such as AD and other age-related disorders^[29]^. Understanding of the relationship between brain aging and the microglia may provide insights into how to prevent unhealthy brain aging.

### Age-related morphological changes in microglia

Throughout the process of aging, the microglia mainly undergo at least two types of morphological changes: hypertrophic microglia and dystrophic microglia. The former is featured with an enlarged cell soma and thickened length of processes, while the latter is characterized by a small body and deramified processes. Hypertrophic microglia, also namely the primed microglia, describe the activated microglia in the brain, which display the increased responses to immune stimuli and lead to an augmented inflammatory activity. A recent study revealed that the total number of microglia and the percent of hypertrophic microglia were age-dependent^[30]^. Additionally, according to a study of postmortem brain tissue within the aphasia variant of AD, hypertrophic microglia were distributed inhomogeneously across the brain and were more commonly distributed in an atrophied grey matter, compared with the resting microglia^[31]^. Compared with hypertrophic microglia often seen following diverse neurology diseases, dystrophy microglia have been suggested as a form of microglial senescence.

**Figure 2:**
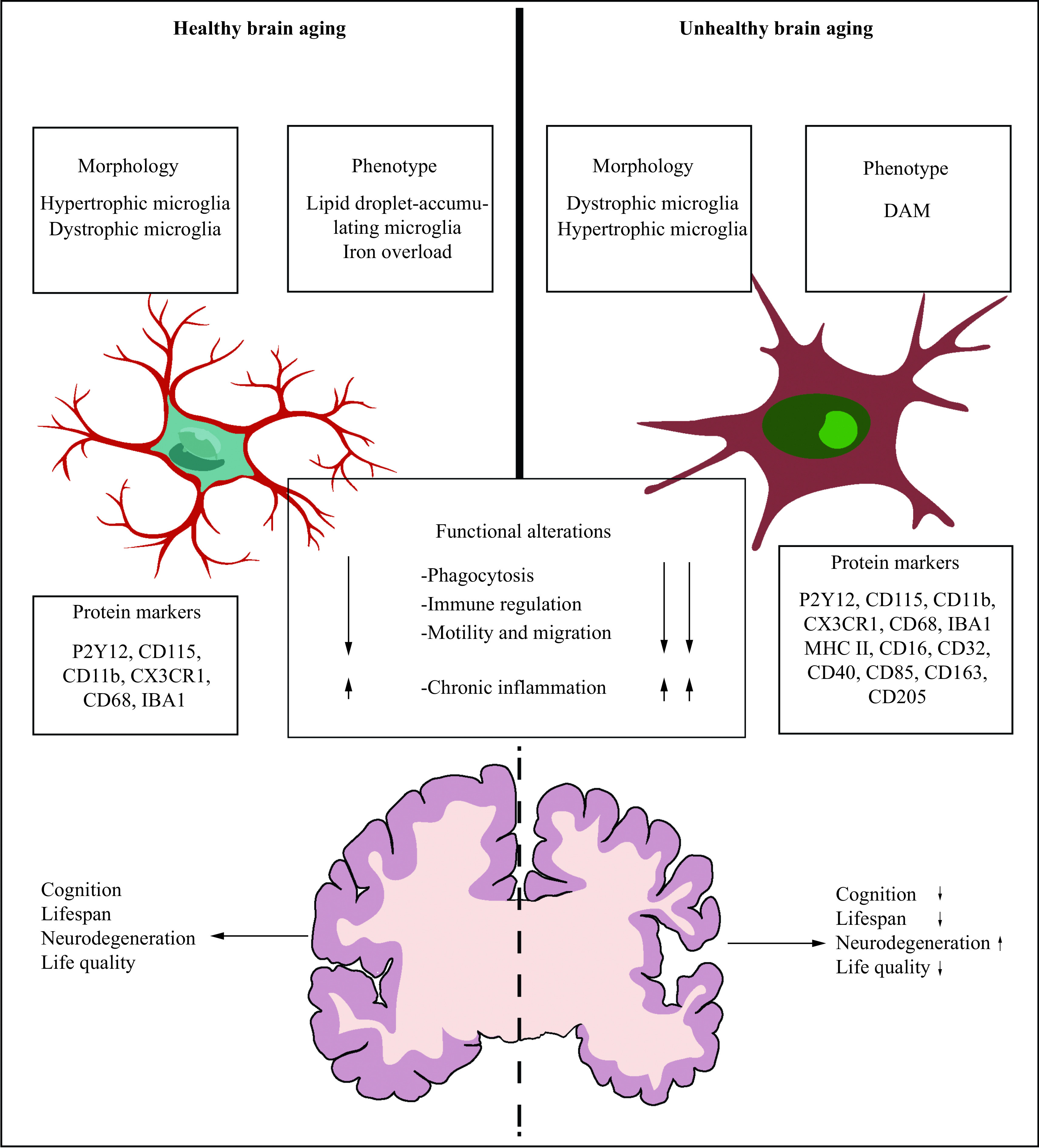
Microglial changes in healthy and unhealthy brain aging.

Dystrophic microglia are distinct from hypertrophic microglia and are a common group of generously identified microglia in the elderly^[26]^. Shahidehpour *et al*^[30]^ performed a series of autopsies and found the increased levels of dystrophic microglia in the aged brain, which were also more common in older brains with neurodegenerative diseases, suggesting that dystrophic microglia are associated with neurodegenerative diseases rather than healthy aging. However, Cárdenas-Tueme *et al*^[32]^, using C57BL/6 male mice at 2, 12, and 20 months, found that the aging led to an increasing proportion of dystrophic microglia in the left medial entorhinal cortex, which resembled a "surveillance state" characterized by the extended processes capable of contacting cells and structures; at the same time, the aged male mice appeared to have a decreasing number of hypertrophic microglia in the fornix, accompanied by damage-associated molecular patterns featured with the downregulation of several microglial subtypes; in addition, based on further biological modeling, they also found that the age-related increases in the fornix volume were associated with dystrophic microglia, thereby contributing to cognitive decline. Taken together, the morphological changes caused by aging are not absolute, and hypertrophic microglia and dystrophic microglia can both exist in an aging brain. These age-related changes in morphology vary according to the region of the brain and are associated with brain function (***Fig. 2***).

In summary, this issue deserves particular investigation, considering morphological plasticity of the microglia is accompanied by functional changes. Understanding how morphological changes in the microglia lead to neuronal dysfunction may provide new insights into the causes of cognitive impairment.

### Age-related function changes in microglia

Mounting evidence suggests that the microglia store iron as aging, but the microglia are not the main iron-storing cell type in the brain^[33]^. Iron, playing a crucial role in neuronal respiration, myelin synthesis, production of neurotransmitters, synaptic plasticity, and metabolic activities^[34]^, can mediate the production of ROS and the formation of lipid peroxides, and thus contribute to aging-related dysfunction and neurodegenerative diseases^[35]^. In aged mice, the inflammatory environment increases non-heme iron levels in the brain through an increased expression of divalent metal-ion transporter-1 (DMT1) and a decreased expression of ferroportin, leading to iron accumulation. The iron overload caused by microglial heme oxygenase-1 (HO-1) overexpression can induce ferroptosis, eventually leading to cognitive declines^[35]^. Additionally, in the postmortem of AD patients, Kenkhuis *et al*^[36]^ found that the microglia took up iron and influenced their functional phenotypes, and these investigators also identified a subset of the microglia with the increased expression levels of the iron storage protein ferritin light chain (FTL) and the ionized calcium-binding adapter molecule 1 (IBA1) but with the decreased expression levels of the transmembrane protein 119 (TMEM119) and recombinant purinergic receptor P2Y, G protein-coupled 12 (P2RY12) (***Fig. 2***); besides, this type of microglia could be seen in patients with the elevated Aβ and tau loadings. Therefore, iron overload increases the degenerative susceptibility of the microglia, leading to oxidative damage, particularly in elderly brains. Likewise, the microglia can build up lipid droplets with aging, which is identified as lipid droplet-accumulating microglia by the RNA sequencing (RNA-Seq) analysis, featuring with phagocytosis defect and an increased production of ROS as well as a higher secretion of inflammatory cytokines (***Fig. 2***). This type of microglia represents a pro-inflammatory and dysfunctional conditions within the aging brain, also contributes to age-related and genetic forms of neurodegeneration^[37]^.

**Figure 3:**
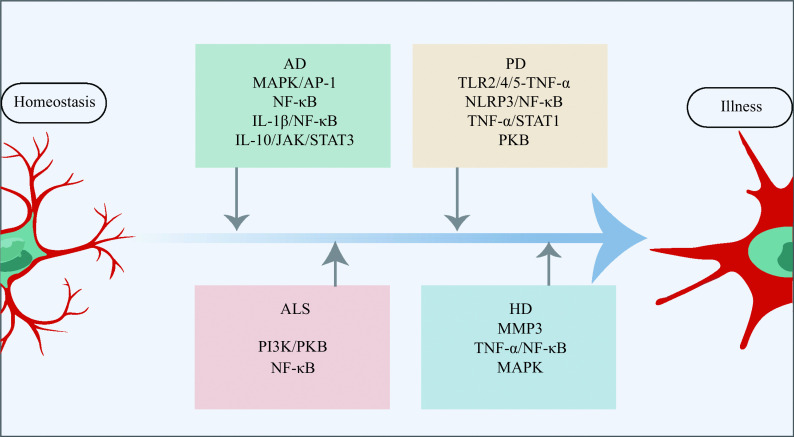
Microglial signaling pathways activated in different neurodegenerative diseases.

With brain senescence progression, the microglia undergo profound differentiation function changes, which is considered a "primed" state, and become hyperactive in response to immune challenges^[38]^. Moreover, these microglia are jeopardized in their phagocytosis activity, along with high levels of pro-inflammatory cytokines, such as tumor necrosis factor-α (TNF-α), interleukin-1β (IL-1β), and interleukin-6 (IL-6)^[39]^ (***Fig. 3***). They undergo replicative senescence, characterized by shortening of telomeres^[40]^, accumulation of lipofuscin^[41]^, reduction of motility, lower phagocytic response^[42]^, *etc*. These microglial modifications are suggested to play a vital role in age-related chronic inflammation and functional declines in the aging brains^[43]^.

### Age-related genomic, epigenomic, and transcriptomic changes in microglia

Recent studies of the single-cell analysis, single-nucleus, and spatial transcriptomic technologies, which provide a clearer understanding of the epigenetic microglial landscape, have renewed our understanding of the variety and heterogeneity of the microglia. According to these studies, microglia undergo age-related changes in genes associated with innate microglial immune activation. They are feathered with a decreased expression of homeostatic genes, such as runt-related transcription factor 1 (*RUNX1*), interferon regulatory factor 8 (*IRF8*), and Spi-1 protooncogene (*PU1*), and an increased expression of immune- and inflammation-related genes encoding numerous complement components and cell surface receptors, as well as genes coding for Fcγ receptors and human leukocyte antigens Ⅰ and Ⅱ^[22]^. Olah *et al*^[44]^ used human brain autopsies and RNA-Seq to conduct a comprehensive transcription landscape assessment of the aged human microglia, and found that some susceptibility genes associated with late-onset AD, such as triggering receptor expressed on myeloid cells-2 (*TREM2*), inositol polyphosphate-5-phosphatase D (*INPP5D*), membrane spanning 4-domains A4A (*MS4A4A*), sortilin related receptor 1 (*SORL1*), and myeloid cell surface antigen 33 (*CD33*), were involved during the aging. Patel T *et al*^[45]^ found that the age-related shifts in the transcriptome of microglia in both mice and humans showed an upregulation of gene transcripts linked to brain inflammation. The lipid droplet-accumulating microglia of aged mice, as previously suggested, had distinct transcriptional states that were regulated by variations of solute carrier family 33 member 1 (*Slc33a1*), lysosomal/endosomal transmembrane protein 3 (*Cln3*), vacuolar sorting protein 35 (*Vps35*), sorting nexin 17 (*Snx17*), Niemann-Pick disease type C intracellular cholesterol transporter 2 (*Npc2*), and granulin precursor (*Grn*) genes^[37]^. These genes can induce autosomal dominant forms of neuropathology. These findings further reinforce the earlier notion that microglia changes in aged brains lose their intrinsic actionability of function regulation, which is associated with aging-related diseases like AD and amyotrophic lateral sclerosis (ALS).

MicroRNAs (miRNAs) participate in gene regulation at the post-transcriptional level and are small (0–22 nucleotides), single-stranded RNAs associated with cancer, aging, and a number of neurodegenerative diseases. MicroRNA expression changes with age appear to be correlated with microglial function/dysfunction, polarization, and regeneration^[46]^. For example, miRNA-146a, by suppressing nicotinamide phosphoribosyl transferase (NAMPT) and inactivating nicotinamide adenine dinucleotide/sirtuins (NAD^+^/SIRT), acts as a modulator of inflammation and the innate immune response, therefore possibly participating in the brain aging^[47]^. However, Liang *et al*^[48]^ discovered that miR-146a overexpression in the microglia inhibited neuronal death, lowered cognitive deficits, switched microglial phenotypes, reduced pro-inflammatory cytokines, and enhanced phagocytosis in precursor protein/presenilin-1 (*App*/*Ps1*) transgenic mice. Therefore, the function of miRNA-146a in human physiology is not yet well understood.

miR-34, on the other hand, is thought to perform a tumor-suppressive role because of its synergistic effect with the widely known tumor suppressor p53^[49]^, and plays a vital role by downregulating genes involved in neuronal survival, synapse formation and plasticity, mitochondrial function, antioxidative system, and energetic metabolism^[50]^. In Drosophila, the absence of miR-34 caused the dysregulation of protein translation and protein cycle in aged brain, causing neurodegeneration, and an accumulation of the repressive histone mark H3K27me^[51]^. miR-34 also suppresses polycomb repressive complex 2 (PRC2) to control chaperone expression and promote healthy brain aging^[52]^. Additionally, miR-29a and miR-29b appear to have neuroprotective effects and cause excessive microglial inflammation, both of which are negatively correlated with mRNA levels of C-X3-C motif chemokine receptor 1 (*CX3CR1*) and insulin-like growth factor 1 (*IGF1*)^[53]^.

By applying these techniques to the understanding of microglial functions in various illnesses, we are becoming aware of the vast variability of microglia populations in both normal and aging brains. Despite the amount of data gathered from studies, these molecules are still poorly understood and further research is necessary to gain insights into their functions in the aging brains of animals and human beings.

## Microglial cells in unhealthy brain aging

Neurodegenerative diseases are characterized by ongoing nervous system damage that causes symptoms in our motor skills and cognitive deficits^[54]^. The prevalence of neurodegenerative diseases is expected to rise with an increasing life expectancy in most countries^[55]^. By the year 2050, dementia is expected to afflict over 130 million people, up from the present 50 million people^[56–57]^. Aging is an independent and leading risk factor for neurodegenerative disorders^[28]^. Therefore, neurodegenerative diseases are considered the results of "unhealthy brain aging". Presently, there are no medicines or other treatments to halt or reduce the progression of these diseases, despite the huge financial investment and millions of suffering people globally^[54,58]^. Historically, neurodegenerative diseases were considered neuron-autonomous disorders. However, the pivotal role of glial cells, such as microglial cells, in disease pathogenesis has slowly come into the focus. In the following sections, we will concentrate on the role of microglial cells in unhealthy aging brains and provide a new perspective and therapeutic strategy for neurodegenerative diseases.

### Microglia in AD

AD is the most common neurodegenerative disease and the leading cause of dementia^[59]^. A recent study conducted in the United States found that around 10.7% of those aged 65 and older suffered from AD^[59]^. The affected individuals typically exhibit prominently progressive cognitive defects, but fewer individuals frequently have nonamnestic cognitive impairment, including deficiencies in expressive speech, visuospatial processing, and executive capabilities^[59]^. Etiologically, the buildup of Aβ peptides released by neurons and the hyperphosphorylated tau in neurons are hallmark pathologies of AD^[60]^.

Recently, microglia have been implicated as a major contributor to AD^[61–62]^. The accumulation of Aβ and tau fibrillary tangles activate the microglia; however, the microglia are also involved in the spread of hazardous proteins^[63–68]^. Using the 18 kDa translocator protein positron emission computed tomography (TPSO-PET) imaging in AD patients, Hamelin *et al*^[69]^ found that TSPO expression in the temporoparietal cortex was greater in AD patients than in controls at both prodromal and demented stages. The 18 kDa TSPO, also known as the peripheral benzodiazepine receptor, is dramatically upregulated in response to microglial cell activation, but expressed at a much lower level in the physiological brain. Therefore, the expression of TSPO is generally believed to typify neuroinflammation^[70]^. In another study, investigators found that an increase in TSPO expression over time was positively associated with three clinical outcomes, including clinical dementia dating, mini-mental state examination, and hippocampal atrophy, suggesting that the increasing neuroinflammation is associated with AD progression^[71]^. Transcriptomic studies of AD mouse models have revealed that the disease process is paralleled in the microglia by a gradual transition from homeostasis to a disease-associated state, which is characterized by the downregulation of homeostatic genes and the expression of known Alzheimer's genes, such as *TREM2*^[72]^. TREM2, a macrophage cell surface receptor, is primarily upregulated by brain microglial cells. According to a recent study, a TREM2 response occurs early in the amyloid cascades^[73]^. Several rare variants in *TREM2*, such as TREM2^R47H^, have emerged and significantly increased the risk by 2- to 4-fold, comparable to the increased risk associated with having one copy of APOEε4, which impairs ligand binding and curtails microglial activation in humans and AD mouse models^[74]^. From a recent autopsy-confirmed cohort, Kim *et al*^[75]^ found that the TREM2 variant was more frequently associated with non-amnestic clinical syndromes and contributed to clinical and pathologic AD heterogeneity by altering the distribution of neurofibrillary degeneration and tau-dependent microglial dystrophy, resulting in hippocampal-sparing and non-amnestic AD phenotypes. All the above evidence demonstrates the function of TREM2 and implies the role of microglial cells in AD pathogenesis^[76]^. However, the progression of AD, such as Aβ accumulation and synaptic dysfunction, tau fibrillation, microglia activation, and neurodegeneration, is not necessarily linear^[77]^. Early microglial activation may have a protective effect against AD^[69,78]^, and the emergence of a chronic inflammatory milieu may be also associated with AD pathogenesis. Therefore, there is a model of neuroinflammation that may explain AD development. That is, the context of chronic inflammation coupled with aging offers an initial stimulus and activates microglial priming, which plays a protective function at the preclinical stage of AD as Aβ deposition emerges. In the latter stages of AD, an invalid clearance of Aβ and tau aggregation hinders microglial phagocytosis and sustains detrimental microglial activation^[62]^.

These studies strongly support the notion that microglial activation may be implicated in both beneficial and detrimental effects, depending on the severity and timing of the disease, individual susceptibility, and prior baseline inflammation. Future studies are needed to answer this question that may be the key to the timing of anti-inflammatory treatments.

### Microglia in Parkinson's disease (PD)

PD, which is clinically characterized by bradykinesia, resting tremor, stiffness, and postural instability, is the second most prevalent neurodegenerative disease, affecting more than 1% of people under the age of 65^[79–80]^. PD is pathologically characterized by the development of α-synuclein aggregations known as Lewy bodies. Dopamine (DA) neurons in the substantia nigra pars compacta are the principal contributors to these characteristics^[81]^. PD is now recognized as a multi-system condition, not just a movement disorder, with considerable immunological and neuroinflammation dysfunction that has been associated with the development of many non-motor symptoms, including gastrointestinal dysfunction and rapid eye movement sleep disorder. Through the use of TSPO-PET, Gerhard *et al*^[82]^ found that microglial activation was greater in PD patients than in healthy controls, suggesting a connection between microglial activation and PD. Duffy *et al*^[83]^ discovered that α-synuclein might activate microglial cells through the toll-like receptor 4 (TLR4) route or the TLR2 pathway (***Fig. 3***), and that microglial cells, in turn, caused injury to dopaminergic neurons. Lavisse *et al*^[84]^ found that microglial activity in the putamen, frontal cortex, and midbrain did not correspond with the severity of motor symptoms or the length of the illness in PD patients. These findings infer that early disease-related microglial overactivation is harmful rather than having a compensatory effect, such as promoting brain inflammation and neuronal damage. The NOD-like receptor thermal protein domain associated protein 3 (NLRP3) inflammasome signaling complex is a multiprotein inflammatory signaling complex involved in the induction of a pro-inflammatory state^[85]^. Reportedly, α-synuclein activates NLRP3 inflammasome signaling in the microglia, and different α-synuclein species lead to distinct microglial responses *via* TLR2 and TLR5 ligation in PD models^[86]^ (***Fig. 3***). Furthermore, the microglia with the accumulated α-synuclein exhibit phagocytic fatigue as well as an overabundance of oxidative and proinflammatory chemicals, leading to the selective degradation of DA neurons and the recruitment of peripheral immune cells^[87]^, which may potentially promote neuroinflammation and speed up the development of PD.

Currently, more investigators have recognized that gut microbiota play a critical role in modulating the physiology of PD *via* the "gut microbiota-brain axis"^[88–90]^. Gut microbiota dysbiosis overstimulates the innate immune system, coupled with a higher intestinal barrier permeability, may provoke neuroglial activation and lead to brain inflammation, ultimately triggering the development of alpha-synuclein pathology^[91]^. Fecal microbiota transplantation, which can reduce gut microbial dysbiosis, reduces the activation of microglial cells and astrocytes in the substantia nigra, and lowers the expression of TLR4/TNF-α in the gut and brain, regulating motor deficits^[92]^. Medicinal plants as anti-inflammatory agents regulating neuroinflammation are effective in PD animal and cell models^[93–94]^, such as mucuna pruriens^[95–96]^, ursolic acid^[97]^, chlorogenic acid^[98]^, Withania somnifera^[99]^ and berberine^[100]^, which may potentially relieve neuroinflammation and slow down the development of PD.

In summary, determining whether the microglia are a cause or an effect of PD, or both, and at what time the microglia may function as an amplifier to produce vast and permanent neuronal DA loss, will be of tremendous importance. However, targeting inflammatory pathways involved in PD treatment needs additional investigations.

### Microglia in Huntington's disease (HD)

HD is an autosomal dominant disorder genetically defined by an increase of trinucleotide (CAG) repeats in the Huntington gene (*HTT*)^[3]^. Since the amino acid glutamine is encoded by CAG, the expansion results in gradual neuronal malfunction and neuronal death, especially in the brain and striatum. As a result, people with HD often exhibit cognitive impairment, mental abnormalities, motor dysfunction, and behavioral disorders^[101]^. Despite extensive investigation into its root cause, the processes behind HD's dominant symptoms, *i.e.*, the selective malfunction and death of neurons, remain poorly understood. Since the early postmortem investigations, neuroinflammation in HD has been documented because of reactive microglia. The expression of defective mutant Huntingtin in the microglia leads to their activation, and the activation of microglial cells leads to neuronal death *via* the activation of death receptors, resulting in oxidative stress^[102]^. In a cross-sectional clinical study by using the TPSO-PET scan and structural magnetic resonance imaging, Rocha *et al*^[103]^ found that the activation of microglia cells in basal ganglia areas involved in HD pathogenesis was enhanced, along with a disease burden, compared with healthy controls. They also found that microglial activation became evident only after the onset of motor symptoms, presented in presymptomatic gene carriers, suggesting that neuroinflammation contributes to illness progression rather than disease initiation; nonetheless, this study had a key disadvantage in that the sample size was particularly small^[103]^. In a rodent study, Savage *et al*^[104]^ observed that, compared with the control group, striatal microglia of HD patients showed age-related decreases in synaptic contact and increases in phagocytosis, suggesting that the microglia may have a direct involvement in synaptic modification and loss, which contributes to HD pathogenesis. Matrix metallopeptidase 3 (MMP3), which is released by neurons, was found to be elevated in HD patients and associated with the patients' motor scores^[105]^ (***Fig. 3***). The elevated MMPs promote microglia activation and release cytotoxic proinflammatory chemicals in the HD brains.

These investigations provide evidence that microglia-mediated neuroinflammation affects the course of HD in different ways, suggesting it may be an oversimplification to consider the microglia as playing only a negative or positive function. Hence, it is undoubtedly desirable to continue exploring possible neuroprotective abilities of the microglia to potentially develop both preclinical and clinical therapies.

### Microglia in amyotrophic lateral sclerosis

ALS is a systemic disorder characterized by a progressive lack of pain and strength in the muscles because of the loss of upper and lower motor neurons in the brain and spinal cord, along with behavioral changes and cognitive decline^[106]^. ALS is also a common neurodegenerative disorder, affecting approximately 4.42 per 100000 population worldwide^[107]^, with a high mortality and disability. The mechanisms underlying selective neuronal malfunction and death of ALS remain unknown^[108]^. A growing body of evidence suggests that microglial-mediated neuroinflammation plays a role in the etiology of ALS^[109]^. Using the high-throughput RNA-seq on postmortem frontal cortex tissue, Dols-Icardo *et al*^[110]^ discovered a coordinated rise in microglial transcripts and the overexpressed neuroinflammatory markers in ALS patients. Therefore, it is clear that microglial cells are involved in the pathogenesis of ALS.

Alshikho *et al*^[111]^ used structural magnetic resonance imaging and TPSO-PET to detect microglial activation and discovered that microglial activity was associated with motor signs, cortical shrinking, the decreased fractional anisotropy, and higher diffusivity. After over six months, they repeated the image examination and found that there was no change in the images of individuals, despite clinical progression. Altogether, these data suggest that microglial activation is present in the early, rather than in the late stage. Moreover, microglial activation was correlated with neuronal and synaptic loss, as well as a quick development of motor and extra-motor illness^[112–113]^; however, it is unclear whether these connections are causative or a result of the accelerated pathology^[114]^. Therefore, more investigations and improved tools are needed to fully characterize how the microglia-mediated inflammatory response occurs at different phases of ALS and where potential therapeutic interventions may be taken to delay disease progression and bring the patient functional recovery.

### Senescent microglia and brain aging

Cellular senescence, defined as a cellular condition, in which the expression of the cyclin-dependent kinase inhibitors, such as cyclin dependent kinase inhibitor 1A (CDKN1A, also known as p21^CIP1^) and/or cyclin dependent kinase inhibitor 2A (CDKN2A, also known as p16^INK4A^) causes an irreversible cell-cycle arrest, is evident in aging, which has been largely seen in cancer and aging investigations^[1,115]^. Senescent cells have been found to multiply with age and in many other diseases, producing a characteristic inflammatory secretome that may lead to illness^[116–117]^.

The microglia, as long-lived cells in the CNS^[118]^, have also been observed to age, with substantial changes in distribution, shape, and behavior^[119]^, including deramification and aberrant swellings in surviving processes, process retraction and cytoplasmic fragmentation, an increase in number/density, and a decrease in distribution regularity^[120]^. Furthermore, as the brain ages, senescent microglia undergo unique molecular changes. In contrast to cells from younger adult mice (2 months old), senescent microglia in the aged mouse brain (1.5 years old), as detected by using "high-dimensional single-cell proteomic mapping" techniques, displayed greater levels of phagocytosis linked to the CD11c and CD14 markers, lower levels of microglial homeostatic checkpoint indicators, including sialic acid-binding immunoglobulin-like lectin H (SIGLEC-H) and CX3CR1, and higher levels of activation markers CD86 and CD44 as well as the inhibitory ligand-programmed death ligand 1 (PD-L1)^[121]^. These changes at the molecular level are consistent with functional features.

Notably, the fraction of microglial cells expressing CD11c and CD14 was observed in old mice, whereas it was almost nonexistent in young mice^[121]^. These findings indicate distinctions between senescent and youthful microglia, but it remains challenging to differentiate them from an "activated" inflammatory state, since they share so many traits, such as an inflammatory secretome that may contain TNF-α, IL-1, and IL-6. To resolve this issue, the International Cell Senescence Association has proposed a suggestion for identifying the existence of senescence traits, such as cell-cycle arrest, macromolecular damage, dysregulated metabolism, and the generation of a senescence-associated secretory phenotype (SASP)^[122–123]^. The SASP includes the production of matrix metalloproteinases, ROS, cytokines, chemokines, and nitric oxide^[122–123]^ that are known to promote microglial activation.

The microglia are profoundly affected by brain aging at the genomic, epigenetic, transcriptomic, and proteomic molecular levels. Reactive microglia have been proven to exist in the brains of elderly people, increasing over time^[124]^. Using a spatial transcriptomics-based method, Kiss *et al*^[125]^ found that brain aging was correlated with increased cellular senescence in the white matter, hippocampi, and cortical grey matter of mouse brains. They performed the gene ontology enrichment analysis and identified the production of pro-inflammatory genes in the activation of microglia, indicating that senescent cells probably play a paracrine role in the genesis of neuroinflammation. The single-cell analysis conducted recently by Hammond *et al*^[126]^ has shed new light on microglia proliferation, showing that the microglia were transcriptionally highly diverse in aged mice, compared with the limited heterogeneity of the microglia during adulthood. Among these, the inflammatory microglia expressed inflammatory signaling molecules, such as chemokines C-C motif chemokine ligand (CCL) 3 and CCL4, cystatin F (CST7), and IL-1β. An additional microglial state, defined by the increased expression of interferon-related genes, such as interferon-induced transmembrane protein 3 (*Ifitm3*) and receptor transporter protein 4 (*Rtp4*), became prominent in the elderly mice brains as well^[126]^. Such features of senescent microglia have been associated with the emergence of neuropathological conditions, including AD/PD.

Senescent microglia also contribute to brain aging. The induction of senescence in the microglia loses the intrinsic machinery that regulates their proper function, including their ability to protect neurons, monitor their surroundings, move, and react to damage. Biochemically, the aged microglia generate more SASP-related molecule-like inflammatory cytokines and ROS. Overproduction of proinflammatory mediators sensitizes the microglia, resulting in an enhanced ineffective response to inflammatory stimuli. Such dysregulated microglia provide inflammatory milieu and have a deleterious effect on neuronal function, affecting synaptic connectivity, jeopardizing white matter integrity, and contributing to normal brain aging deterioration and neurodegeneration.

By using single-cell and nuclear RNA-seq, Ogrodnik *et al*^[127]^ demonstrated that p16^INK4A^ expression raised with age in several cell types, although the microglia were more visible than other cell types, and that only p16^INK4A^-positive microglia could be eliminated by pharmacogenetics and pharmaceutical treatments targeting senescent cells. In a tau-based neurodegenerative model, destroying senescent (non-dividing) microglial cells significantly decreased age-related brain inflammation and cognitive impairment in mice^[128]^.

Therefore, it is reasonable to suspect that the elimination of senescent microglia may help treat neurodegenerative illnesses. However, this has not yet been conclusively established. It also remains unknown whether the development of aging-related microglia results from the CNS environment that perceives changes or from the loss of intrinsic molecular machinery that regulates appropriate functioning throughout senescence. To find answers to these key questions in the field, investigators will need to specifically identify and target these aged microglia.

## Conclusions

Currently, we live in an aging society. Dealing with the effects of the aging population is a great challenge. One of the biggest issues associated with aging is the deterioration of brain function. Cognitive decline correlated with brain aging is on the rise, hence increasing the risk of neurodegenerative illnesses. Dual roles of the microglia in brain aging have been demonstrated in this review. However, the regulation mechanism and its relevance to age-related disorders remain unclear, and potential issues with artificially augmenting microglia have not been investigated. Considering the key role of microglial cells in age-related illnesses, it is extremely probable that new promising techniques may offer potential therapies for age-related diseases.
